# Presence of Coliforms and Reduced Water Quality in the Second Biggest Reservoir in São Paulo, Brazil

**DOI:** 10.3390/life15050729

**Published:** 2025-04-30

**Authors:** Andrezza Nascimento, Lorena A. Fernandes, Carlos A. O. de Biagi, Marta A. Marcondes, Sabri Saeed Sanabani

**Affiliations:** 1 Post-Graduation Program in Translational Medicine, Federal University of São Paulo, São Paulo 04038-901, Brazilmarta.marcondes@online.uscs.edu.br (M.A.M.); 2 Department of Pediatric Oncology, Dana Farber Boston Children’s Cancer and Blood Disorders Center, Boston, MA 02115, USA; 3 Broad Institute of MIT and Harvard, Cambridge, MA 02142, USA; 4 Project IPH, Municipal University of São Caetano do Sul, São Caetano do Sul 09521-160, Brazil; 5 Laboratory of Medical Investigation LIM-56, Division of Dermatology, Medical School, University of Sao Paulo, Av. Dr. Enéas Carvalho de Aguiar 500, Sao Paulo 05403-000, Brazil; sabyem_63@yahoo.com; 6 Laboratory of Medical Investigation LIM-03, Division of Pathology, Medical School, University of Sao Paulo, Sao Paulo 05403-000, Brazil

**Keywords:** Guarapiranga reservoir, bacteriome, 16S rRNA, ARGs, water quality, coliforms

## Abstract

(1) Background: The Guarapiranga reservoir, located in the metropolitan region of São Paulo (RMSP), plays an important role in supplying water to the population. However, the growing urbanization in the region, which has occurred in a disorderly manner and lacks basic sanitation infrastructure, has had a detrimental impact on the reservoir’s conditions. The aim of this study was to evaluate the physicochemical parameters and detect coliforms to determine the water quality of the Guarapiranga reservoir, as well as to characterize the microbial diversity and antimicrobial-resistance genes (ARGs) present in the reservoir water. (2) Methods: Four sampling campaigns of the Guarapiranga reservoir were carried out between October 2020 and July 2022. Physicochemical analyses, and selective microbiological culture for coliforms, as well as the extraction of bacterial DNA for subsequent sequencing and search for ARGs were carried out. (3) Results: Analysis of the physicochemical results showed a progressive reduction in the quality of the reservoir’s water, and the microbiological tests consistently showed the presence of *Escherichia coli*, *Salmonella* spp., *Shigella* spp. and *Klebisiella* spp. in the water samples collected from the reservoir. Analyses of the sequencing data showed the predominant presence of the phyla Proteobacteria, Cyanobacteria, Bacteroidetes, Verrucomicrobia, Planctomycetes, and 12 ARGs were detected in the reservoir. (4) Conclusions: The increase in sewage discharge, mainly due to the growth of irregular housing, has affected the quality of the water, as indicated by the physicochemical analysis and detection of coliforms and ARGs.

## 1. Introduction

At the beginning of the 20th century, more precisely in 1906, the São Paulo Tramway, Light and Power Company began building the Guarapiranga reservoir to regulate the flow of the Parnahyba hydroelectric plant, built between 1899 and 1901 in the town of Santana do Parnaíba [1]. Two years after it began, the project aimed at generating electricity [2] was completed [1]. However, its construction not only met this practical need but also transformed the surrounding region into a leisure destination, renowned for its stunning landscapes [2]. In 1917, the São Paulo Yacht Club was founded, which was the first yachting club on the reservoir and. Then, in the 1920s, the reservoir was also used to supply water to the São Paulo and Santo Amaro regions [1]. This new status as a recreational area gave rise to the development of farms, hotels, sailing clubs, and even the Interlagos racetrack, boosting real estate values, which led to the creation of luxury subdivisions and valuable rural properties [2].

In the 1980s, algae became a significant concern, affecting both water treatment and energy production [3]. In addition, in the same decade, the water quality and quantity were also compromised due to sewage dumping and diffuse pollution [4]. In the 1990s, the Guarapiranga Program, created by the São Paulo State Government and financed by the World Bank, aimed to restore the environment and improve the quality of life of the region’s population by building water supply and sewage collection networks, as well as re-urbanizing the slums. However, this action did not completely solve the problems [4]. In 1991, in response to an alarming proliferation of algae, São Paulo City Hall and the São Paulo State Basic Sanitation Company (SABESP) launched the Guarapiranga Basin Sanitation and Environmental Recovery Program. This program aimed to restore water quality and implement measures to reverse the negative trends in the region [2].

However, the disorderly occupation, lacking a basic sanitation infrastructure, has had a detrimental impact on the condition of the reservoir [5]. In addition, the exploitation of specific mineral resources, such as clay, mineral water, sand, granite, and kaolin, has emerged as an additional environmental concern in the Guarapiranga reservoir region. This exploitation has resulted in the removal of vegetation and soil, erosion processes, soil and water contamination, as well as a reduction in the water storage capacity [6]. Due to these factors, the Guarapiranga reservoir faces the challenge of eutrophication, which can increase the incidence of ecological and health problems [7].

The Guarapiranga reservoir has played a multifaceted role that goes beyond water supply. Its usefulness extends to water sports, agriculture, and it is home to several industries in its vicinity. The western region of the reservoir is home to both slums and the remarkable Guarapiranga Park, which boasts a rich plant biodiversity [3]. However, exponential population growth, coupled with pollution resulting from sewage dumping and diffuse pollution from homes, businesses, small industries, agricultural activities, among other sources, has triggered an increase in soil erosion, mainly due to the loss of native vegetation for urban expansion, resulting in silting up near the water fountains [4]. In 2013 and 2014, the reservoir was classified as eutrophic or hypereutrophic in most months of those years, due to the large loads of nutrients that were discharged into Guarapiranga by its tributaries [8]. Despite the degradation of the Guarapiranga fount and its surroundings, the Guarapiranga reservoir supplies almost half of the drinking water consumed in the city of São Paulo [1]. According to the São Paulo Government portal, at the end of 2014, Guarapiranga supplied 4.9 million people in the São Paulo Metropolitan Region (RMSP) and, after works aimed at improving the supply to the south of the city of São Paulo, the system’s water production capacity was 15,000 L of water per second [9].

Bacteria are known to be responsible for the processes of mineralization of organic matter and nutrient recycling in aquatic environments, as well as the process of water purification [10]. In terms of water purification, bacteria are important for this process because they remove organic and inorganic contaminants through biological processes. In addition, wastewater treatment systems use bacteria to biodegrade toxic substances in order to improve water quality. In addition to that, bacterial biofilms are used in water remediation technologies to degrade specific pollutants such as hydrocarbons and heavy metals [11].

In the context of public health, some bacteria are beneficial and essential for the healthy functioning of the human microbiome, as they aid digestion, synthesize vitamins and protect against pathogens [12]. On the other hand, some bacteria can cause serious illnesses, such as the contamination of recreational and drinking water with bacterial pathogens such as *Escherichia coli* and *Vibrio cholerae*, which can trigger disease outbreaks. The presence of pathogens in aquatic ecosystems represents a significant risk to public health, especially in areas with inadequate sanitation [13].

Most of the antimicrobials used by humans and animals are excreted in a partially or completely unmetabolized form, and usually with active components, which are discarded in the environment, especially in the water; consequently, the survival of resistant microorganisms can occur [14]. Resistance to antimicrobials can also occur due to the gain of antimicrobial-resistance genes (ARGs), which can be spread to other bacterial populations through horizontal transfer via bacteriophages, plasmids, and transposable genetic elements [15]. However, ARGs can also spread between humans, animals, and environments and, more precisely in the case of aquatic environments, which are prone to contamination from different sources, they can offer a favorable location for the growth of bacteria and the exchange of genetic materials [16], which leads us to believe that aquatic environments can serve as reservoirs for the accumulation of ARGs [16]. Regarding wastewater, it is known that antimicrobials are partially removed during the treatment of this water [17], while the treatment of drinking water with chemical disinfectants, although aimed at inactivating pathogenic microorganisms and preventing microbial growth in the distribution network, can generate by-products with mutagenic and carcinogenic effects, as well as selecting antibiotic-resistant bacteria [18].

Therefore, the resistome encompasses genes commonly present in nature, evolved through the selective pressure of antimicrobials, new resistance genes (anthropo-genic) that have arisen from proto-resistance genes and cryptic genes. All this material is multiplied and propelled within the microbial population through mutations, horizontal gene transfer, and mobile genetic elements for transference between environments [19].

It is known that water contamination can affect public health and the environment. For example, mining waste releases heavy metals and chemical pollutants, contaminating drinking water sources and increasing the risk of diseases, including cancer. In addition to health impacts, contamination compromises agriculture and fishing, affecting the local economy [20], while the contamination of water by radioactive and toxic elements poses a risk to the health of the population and the aquatic ecosystem [21]. Therefore, ascertaining that water is suitable for the various applications used by society requires the evaluation of specific quality indices. In addition, monitoring the parameters and substances present in water is related to the final destination of the water. In this way, physicochemical and biological analyses become important for monitoring bodies of water that are intended for human supply [22]. Therefore, this study aimed to assess the water quality of the Guarapiranga reservoir by analyzing physical–chemical parameters and detecting coliforms, as prescribed by Brazilian legislation. Since this law only mandates basic methods, the study also aimed to evaluate the resistome present in the reservoir’s water, as some ARGs (antibiotic-resistance genes) can indicate anthropogenic water contamination. Additionally, since coliform detection focuses on a limited aspect of microbial composition, the study further aimed to characterize the microbial diversity in the reservoir through next-generation sequencing (NGS) of the bacterial 16S rRNA gene.

## 2. Materials and Methods

### 2.1. The Reservoir and Sample Collection

**Figure 1:**
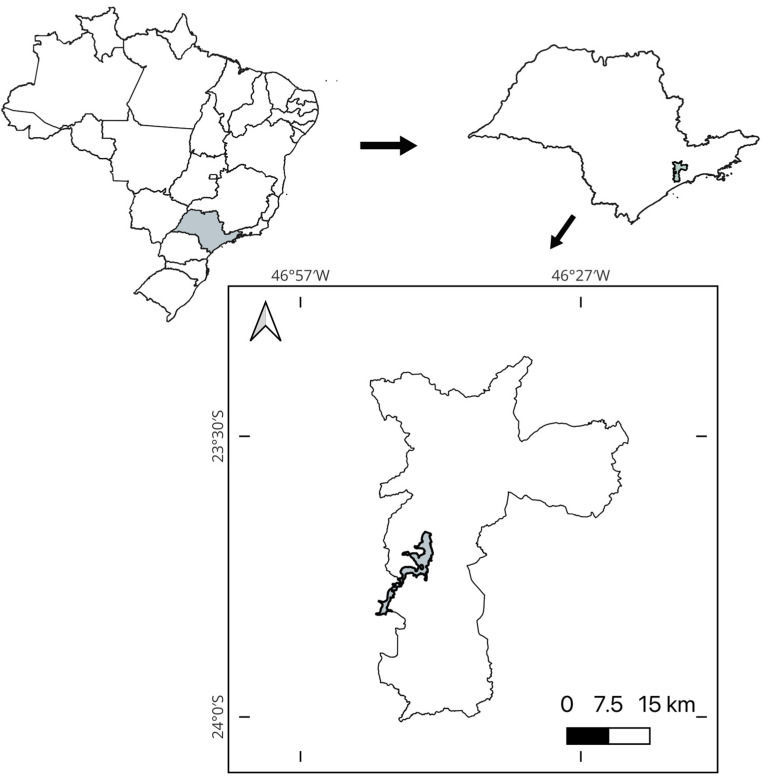
Localization of the Guarapiranga reservoir. The reservoir is in São Paulo city, São Paulo State, Brazil. The map was generated using QGIS 3.38.0-Grenoble.

The Guarapiranga reservoir is located in the state of São Paulo, southeastern Brazil, and is part of the Alto Tietê Hydrographic Basin. The reservoir is very important for supplying water to the São Paulo Metropolitan Region (RMSP) (Figure 1). This reservoir is the second largest source of water for the RMSP and, in 2016, the average flow of the reservoir was 14 m^3^/s^−1^, in a region characterized by an average temperature of 17.5 °C and a climate classified as humid subtropical [23].

During the study, four sampling campaigns were carried out at different times: October 2020, May 2021, September 2021, and July 2022. Ideally, the four collections would be carried out over a period of one year, in the four different seasons. However, due to the difficulty in accessing the boat used for collecting samples during and after the COVID-19 pandemic, the collections did not follow the idealized pattern, but rather according to the availability of the boat.

**Figure 2:**
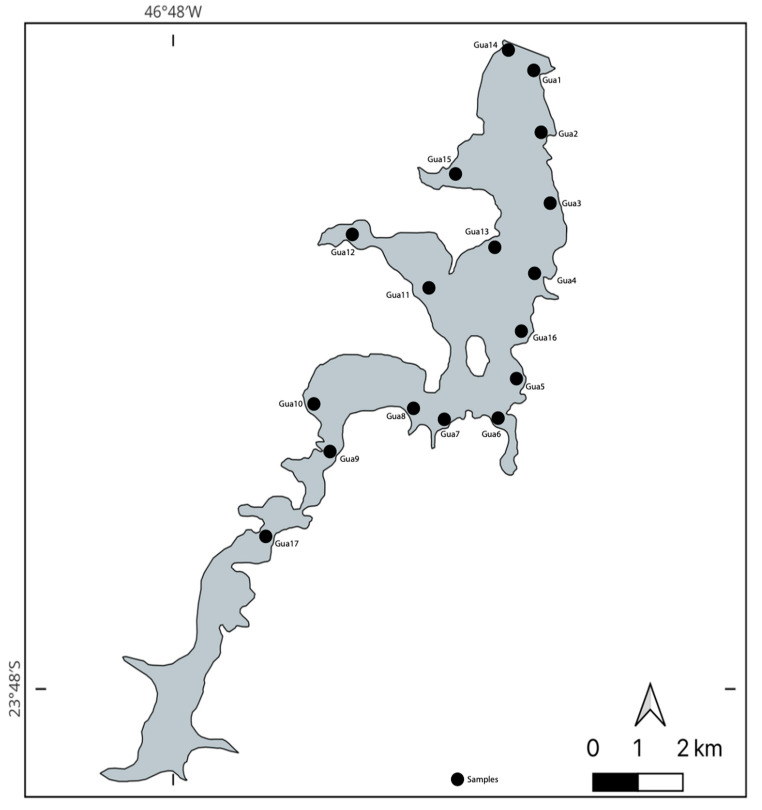
Representation of the sample collection points of the Guarapiranga reservoir. The map was generated using QGIS 3.38.0-Grenoble.

Samples were collected from 17 different points in the Guarapiranga reservoir, based on its the best representation: since the reservoir is in a fully urban area, the 17 sampling points were chosen based on the best representation of each region of the reservoir. For example, regions near major avenues, streets, and highways with heavy traffic, areas with illegal housing in informal settlements, and regions with parks and clubs were included. These points remained the same throughout the four collections. It should be noted that during the first collection, due to the low level of the reservoir, it was not possible to collect from point 17. For each point during the four campaigns, samples were taken from both the surface and the bottom of the reservoir. In short, the samples were taken from the side of a boat, located just below the surface of the water at a depth of 10 cm, as well as from the bottom of the reservoir; the bottom water was collected approximately 0.1 m above the sediment to avoid capturing soil material, following the previously described methods [24]. At each collection event, the Van Dorn sampler was filled with enough water to fill a sterile 250 mL plastic laboratory bottle. All samples were collected in quadruplicates to ensure robust results. Immediately after collection, the samples were refrigerated at 4 °C in a thermally insulated box and then transported to the laboratory; the samples intended for bacterial DNA extraction were stored at −80 °C until genomic DNA extraction was performed. Figure 2 illustrates each point collected during this study, and the Supplementary Table S1 indicates the geographical coordinates of each point.

### 2.2. Physicochemical Analysis

To assess the physical and chemical conditions of the samples, the temperature, dissolved oxygen (DO), and pH of each sample were measured at the collection site using a multi-parameter water meter (HI98494, Hanna Instruments (Barueri, Brazil). In addition, the water samples collected were also subjected to analysis of parameters such as turbidity (Lovibond-Turbcheck), nitrate, phosphorus, and ammoniacal nitrogen (Pastel-Uviline-Spectrophotometers by SECOMAM-Aqualabo Group (Alès, France). Finally, the biochemical oxygen demand (BOD) of the samples from the four surface collections was also evaluated. The instruments were calibrated according to the manufacturer’s standards [25]. Prism 10—version 10.0.0 (131) was used for the statistical analysis of these indices; the data in the table used were of the grouped type, and line statistics were analyzed.

### 2.3. Bacterial Culture

Culture-based methodologies for quantitative and qualitative microbiological analysis of the water samples, with a focus on pathogenic enterobacteria (e.g., *Salmonella* spp., *Shigella* spp. and *Escherichia coli*), were used for all points of the four collection campaigns, both from the surface and the bottom of the reservoir, as described before [26].

Initially, the serial dilution process was carried out, in which the sample was diluted in a series of dilutions, resulting in dilution tubes of 10^−1^ (1 mL of sample was diluted in 9 mL of dilution water, with 1.5% bacteriological peptone), 10^−2^ and 10^−3^. Next, test tubes with lids, inverted Durhan tubes, were prepared containing 10 mL of Lauryl Sulphate Broth (LST), which were prepared according to the manufacturer’s specifications (BIOLOG, Transporte e Logistica (Belo Horizonte, Brazil). Then, 1 mL of the serial dilutions were inoculated in triplicates into tubes of LST broth, a selective medium for coliforms, followed by incubation at 35 °C and subsequent analysis of the positive results after 48 h.

For the determination of CFUs, Plate Count Agar (PCA) was used, a standard microbiological growth medium used for total counting of aerobic and anaerobic heterotrophic bacteria from water samples. The medium was inoculated with dilutions of 10^−1^, 10^−2^ and 10^−3^ in triplicate, and after incubation at 36 °C for 48 h, the CFUs were quantified using a manual colony counter. Gram staining was carried out to differentiate Gram-positive and Gram-negative groups among the CFUs grown. Prism 10—version 10.0.0 (131) was used for the statistical analysis of the CFUs. The Prism settings used were according to this description: the data in the table used were of the grouped type, and the line statistics were analyzed. To create the graphs, the heat map type graph was selected, and the averages of the values for the four samples, both from the surface and the bottom of the reservoir, were plotted for each point.

In addition to characterizing the enterobacteria present in Guarapiranga water, this study also aimed to detect coliforms. To this end, the identification of bacterial groups, specifically enterobacteria, involved several steps. Initially, 5 µL of the suspensions from the positive LST tubes, with gas production and turbidity, were inoculated into Bright Green broth (HiMedia Laboratories Pvt. Ltd., Maharashtra, India), selective for Gram-negative bacteria, used for detecting coliforms and Salmonella non-Typhi and Paratyphi in water, prepared according to the manufacturer’s standards (KASVI—Liofilchem—Italy) and then incubated in a bacteriological oven at 36 °C for 48 h. Subsequent steps included transferring to specific isolation media (Salmonella-Shigella Agar (SS Agar), MacConkey Agar, EMB Agar (Eosin Methylene Blue)), isolation on appropriate agar plates, incubation and identification based on staining characteristics and colony type. Finally, in cases of ambiguity regarding the groups, the colonies were transferred to a Rugai medium with Lysine for the precise identification and confirmation of specific groups of enterobacteria (NewProv—Lot: 18901).

### 2.4. Bacterial DNA Extraction, Amplification of the 16S rRNA Gene, Library Preparation and Sequencing

Regarding the sequencing, only the 16 surface samples collected during the first collection campaign were processed. Initially, a volume of 40 mL of water for each sample point collected was centrifuged at 4000× *g* for 20 min. Then, approximately 50 to 100 μL of the resulting precipitate was transferred to a PowerSoil^®^ DNA Isolation Kit PowerBead tube (MO BIO Laboratories: Carlsbad, CA, USA), containing lysis beads and 750 μL of cell lysis solution. The genomic DNA was then extracted using the MO BIO PowerSoil DNA Isolation Kit (Qiagen Biotecnologia Brasil Ltd., São Paulo, SP, Brazil), following the manufacturer’s standard protocol. The extracted DNA was eluted in 50 μL of MoBio elution buffer and stored at −20 °C until further analysis.

For the amplification of a specific fragment of the 16S rRNA gene covering the V3-V4 regions, the Bakt_341F/Bakt_805R primer set with Illumina adapter sequences was used. The PCR conditions and cycling were previously elucidated by Klindworth et al. [27]. To ensure the purity of the amplified products, a PCR purification kit from Zymo Research (ZR-96 DNA Clean, Concentrator-5 (Deep Well) was used, following the manufacturer’s guidelines.

After purification and quantification with a Qubit 2.0 Fluorometer (Life Technologies: Carlsbad, CA, USA), the amplicons from each sample were combined in equimolar concentrations and subsequently diluted to 4 nM. DNA indexing and library preparation were meticulously carried out following established methodologies [28,29,30]. The prepared library was loaded onto an Illumina MiSeq cartridge (Illumina, San Diego, CA, USA) for fragment sequencing.

### 2.5. Sequencing Analysis—EzBioCloud and Qiime 2

The initial image analysis, base calling, and data quality assessment were carried out on the MiSeq device (San Diego, CA, USA). The generated sequences underwent further refinement and analysis using the 16S Microbiome Taxonomic Profiling pipeline, which was carefully implemented in EzBioCloud [31]. Briefly, next-generation sequencing (NGS) data were submitted to the Microbiome Taxonomic Profiling (MTP) pipeline, using the 16S PKSSU4.0 prokaryote database as a reference, with the target taxon chosen being Bacteria. Pre-processing of the reads involved merging paired reads, removing primers, detecting, and filtering low-quality sequences, and extracting non-redundant reads. The VSEARCH program was then used to search for and calculate similarities between the sequences. The cut-off criterion used for species-level identification was 97% similarity in the 16S rRNA gene. The presence of chimeras was verified using the UCHIME program. The query sequences that passed quality control were compared to the EzBioCloud 16S database for species-level identification, using a cut-off point of 97%. The sequences that did not obtain a 97% match were grouped together using the UCLUST tool, with a similarity threshold of 97%, to create Operational Taxonomic Units (OTUs). The species identified in the first stage and the OTUs from the clustering process were combined to form the final set of OTUs, which was used to calculate the alpha diversity indices. To avoid overestimation, unique sequences were excluded from the OTU selection process.

An analysis flow was also carried out in the Qiime 2 software [32]. Initially, the sequences were subjected to a demultiplexing process, followed by quality control using the DADA2 tool. Next, the phylogenetic tree was built using the Mafft program, which aligns multiple sequences to create a Feature Data artifact in Qiime 2. Subsequently, an alignment filtering procedure was used to remove highly variable positions. The FastTree program was then applied to generate an unrooted phylogenetic tree. Finally, the tree was rooted at the midpoint of the longest distance between the tips of the unrooted tree, using the midpoint rooting method. For the taxonomic analysis, a machine learning module (sklearn) was used in conjunction with the Greengenes database, applying a threshold of 97% to create the Operational Taxonomic Units (OTUs).

Although the two platforms have the same analysis purpose, due to limitations in reproducing the results generated by EzBioCloud, it was decided to keep both analyses in this study, so that the results would be complementary.

### 2.6. Antibiotic Resistance

To comprehensively assess the spread of antibiotic-resistance genes (ARGs), a total of 30 primer sets were selected to profile the resistome of representative Guarapiranga surface water samples (Table 1), as detailed previously [33]. The bacterial 16S rRNA gene was used as a positive control. Therefore, 5 μL of DNA extracted from each of the 16 surface points collected during the first collection campaign were pooled to test positivity or negativity for each resistance gene through the PCR technique.

## 3. Results

### 3.1. Physicochemical Results

**Figure 3:**
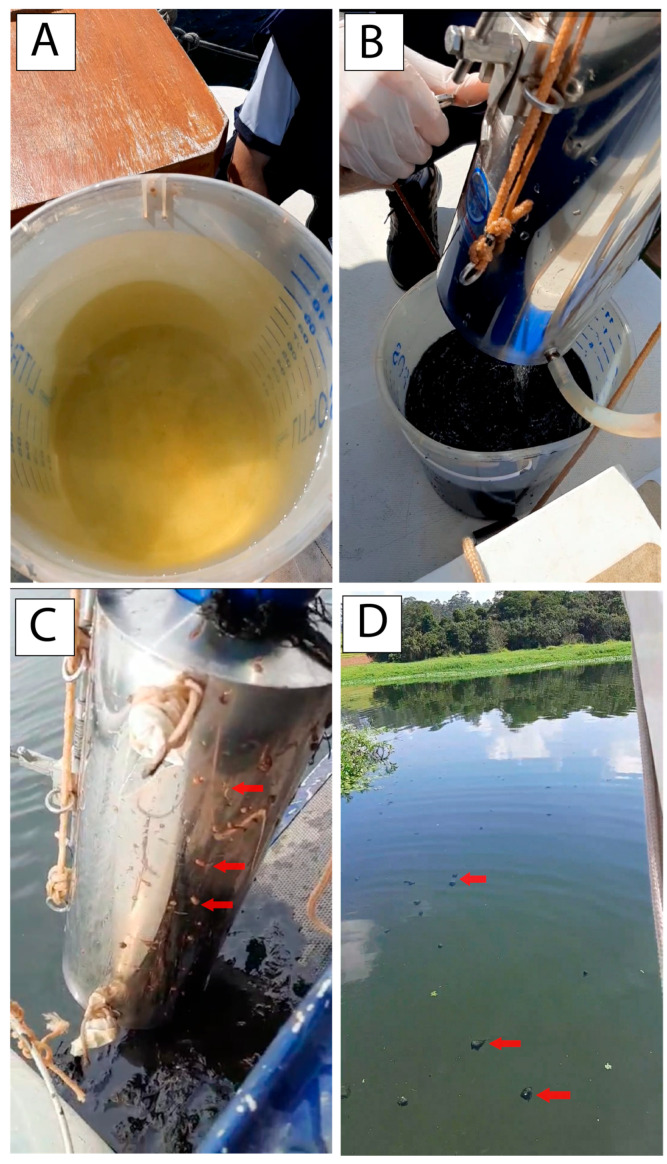
Images taken during the collection carried out in July 2022. (**A**) Water collected from the surface of the reservoir at point Gua6. (**B**) Water collected from the bottom of the reservoir at point Gua6. (**C**) The red arrows indicate the presence of leeches adhered to the outer walls of the bottom collection bottle at point Gua14. (**D**) The red arrows show the fecal plates present at point Gua6.

This study presents a comprehensive analysis of water quality parameters collected during four separate sampling campaigns (October 2020—spring, May 2021—fall, September 2021—transition of winter to spring, and July 2022—winter) in the Guarapiranga reservoir (Figure 3). A total of 134 samples were taken and processed from both the surface and the bottom of the reservoir at 17 designated points.

The reservoir is in São Paulo city, the biggest city in Brazil and South America. Therefore, the collection points Gua1 to Gua3, and Gua16 are close to the big and busy Atlantic Avenue; Gua4 is next to the busy Frederico René de Jaegher Street; Gua5, 6, 10, 13, and 11 are close to golf, tennis, nautical, and soccer clubs—respectively; while Gua7 and 8 are close to the Sacred Ground of Guarapiranga—World Messianic Church of Brazil; Gua9 and 17 are close to the highway Rodoanel Mário Covas; Gua12 is next to the big and busy Jardim Angela district; Gua14 is next to the Guarapiranga Park and to the capitation spot of water for the city supply; and Gua15 is close to the Guarapiranga Ecologic Park. That being said, the water of all the sample collection points showed colors ranging from light green to dark green/black during the four campaigns.

The samples collected were analyzed for turbidity, water temperature, dissolved oxygen, pH, ammonia, nitrate, phosphorus, and biochemical oxygen demand levels (Supplementary Tables S2 and S3).

Turbidity levels in the reservoir showed temporal and spatial variations. The surface turbidity averages were 15.32 NTU (collection 1), 6.98 NTU (collection 2), 27.79 NTU (collection 3) and 6.78 NTU (collection 4). Notably, the lowest and highest surface turbidity values were recorded at Gua10 (2.12 NTU) during collection 2 and Gua1 (46.7 NTU) during collection 1, respectively. On the other hand, the turbidity averages at the bottom were 166.33 NTU, 157.71 NTU, 185.35 NTU, and 139.20 NTU for collections 1 to 4, respectively, with the highest turbidity value of 960 NTU observed at Gua2 during the fourth collection.

Water temperatures showed limited fluctuations over the four sampling campaigns. Average bottom temperatures ranged from 19.72 °C (collection 4) to 21.36 °C (collections 1 and 2). Meanwhile, average surface temperatures ranged from 20.20 °C (collection 4) to 22.32 °C (collection 3).

Dissolved oxygen levels showed varied patterns throughout the study period. The average DO at the surface ranged from 6.85 mg/L (collection 1) to 9.31 mg/L (collection 4), while at the bottom it ranged from 4.18 mg/L (collection 1) to 7.36 mg/L (collection 4). The lowest surface DO value (1.9 mg/L) was recorded at Gua15 during collection 3, while the highest value (19.7 mg/L) occurred at Gua4 during collection 4. For the bottom layer, the lowest DO (0.8 mg/L) was observed at Gua6 during collection 1, and the highest (20.2 mg/L) was recorded at Gua4 during collection 4.

The average pH levels at the surface ranged from 6.88 (collection 4) to 7.13 (collection 1), while at the bottom they ranged from 6.73 (collection 4) to 7.02 (collection 2). Ammonia levels in the surface samples varied on average from 3.61 mg/L to 4.24 mg/L, and in the bottom samples, the average variation was from 4.01 mg/L to 4.71 mg/L. Both nitrate and phosphorus showed lower averages in the surface samples compared to the bottom samples. Regarding the biochemical oxygen demand (BOD), the lowest average was observed at point Gua7 (4.975 mg/L) and the highest at point Gua15 (11.125 mg/L).

### 3.2. Microbiological Culture

**Figure 4:**
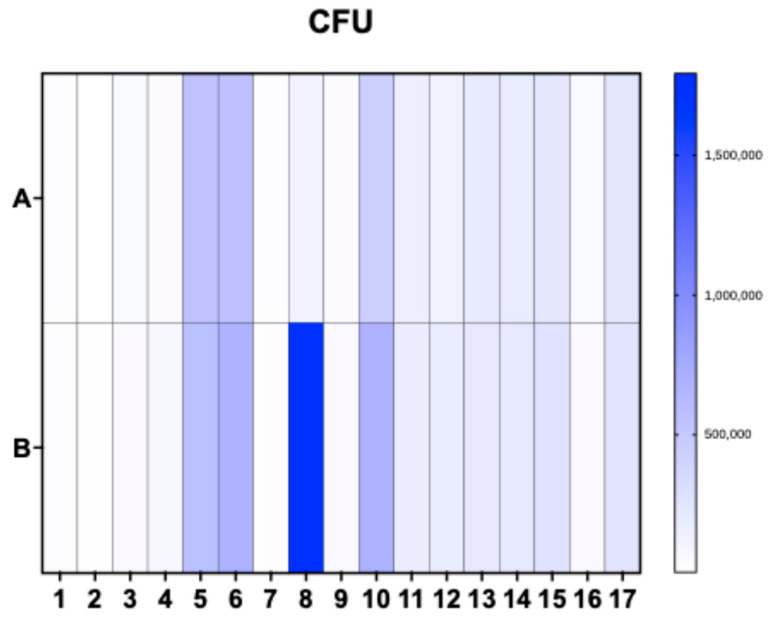
Heat map representing the average CFU count for each point collected from Guarapiranga. (**A**) represents the surface averages, while (**B**) represents the bottom. The numbers from 1 to 17 represent each sample collection point. For each point studied, the samples present similar CFU; therefore, this figure presents the average CFU for each sample studied. Regarding point Gua8, this point is close to the Sacred Ground of Guarapiranga—World Messianic Church of Brazil.

The water samples obtained from each collection point, from both surface and bottom of the reservoir, at the four different times were analyzed using microbiological cultures. The Colony Forming Unit (CFU) (Figure 4) counts resulted in averages of 189,306.25 CFU, 201,017.65 CFU, 190,829.41 CFU, and 144,835.29 CFU, corresponding to each collection from the surface of the reservoir, respectively. From the bottom, the averages were 253,143.75 CFU, 274,511.76 CFU, 255,400 CFU, and 496,882.35 CFU.

The results of the cultures remained consistent throughout all the collections, showing the constant presence of *Salmonella* spp. and *Escherichia coli* at all the points and in all four collections. *Shigella* spp. was identified at 16 of the 17 points sampled, while *Klebsiella* spp. was observed at 10 of the 17 points. It is worth highlighting the importance of point Gua5, as it was the only one with the presence of *Pseudomonas* spp. (Table 2).

### 3.3. Sequencing Data

In this study, next-generation sequencing technology and both EzBioCloud and Qiime 2 software were used to conduct a comprehensive analysis of microbial diversity in the Guarapiranga Reservoir. After applying strict quality control filters in EzBioCloud, 87,099 low-quality amplicons, 121,994 untargeted amplicons and 632,043 chimeric amplicons were removed. Subsequently, a total of 732,398 valid sequences were obtained, with sequence counts ranging from 34,882 (sample Gua12) to 55,704 (sample Gua8), and an overall average of 45,775 sequences per sample. The average size of the sequences was determined to be 452 bp. The analysis of operational taxonomic units (OTU) revealed a wide range of richness, with the smallest OTU containing 1775 sequences (sample Gua12) and the largest containing 3455 sequences (sample Gua6), with an average of 2534 sequences per OTU. In addition, the average coverage of the sequenced data was calculated as 99.22%, with values ranging from 98.3% to 99.7%, indicating a high depth of sequencing and coverage of microbial taxa in the reservoir (Supplementary Table S4).

Using the Qiime software (version 2023.5) to carry out the demultiplexing procedure, it was possible to process a total aggregate of 4,806,909 reads (Supplementary Table S5). This sample ranged in amplitude from 98,725 to 424,017 reads, with an average of 300,431.81 and a median of 308,318 reads. It is also worth noting that the average length of the sequences reached 344.55 base pairs (bp), covering a spectrum between 218 bp (minimum value) and 400 bp (maximum value), with a variation of 182 bp and a statistical dispersion quantified by a standard deviation of 47.86.

In the analytical stage, identification revealed the presence of a total of 1345 distinct Operational Taxonomic Units (OTUs), culminating in a total count of 4056 OTUs. This provided a perceptible and in-depth appreciation of the underlying diversity present in this dataset.

### 3.4. Phylogenetic Analysis

**Figure 5:**
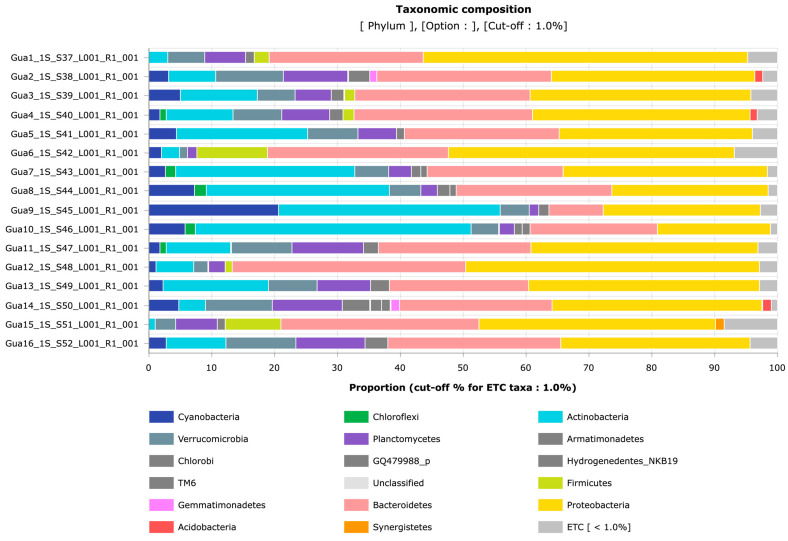
Phylogenetic analysis made through EzBioCloud software (version 202303). The Proteobacteria phylum is predominant at all the 16 sampling points.

On EzBioCloud, the phylogenetic analysis revealed the predominant presence of the phylum Proteobacteria at all the sampling points, along with the phylum Bacteroidetes, except for point Gua9. The phylum Actinobacteria was also significantly abundant at six of the 16 sampling points. On the other hand, the phylum Cyanobacteria was observed in high proportion only at point Gua9 (20.88%). The Planctomycetes and Verrucomicrobia phyla were also identified in all the samples, but in smaller proportions compared to the Proteobacteria phylum (Figure 5).

**Figure 6:**
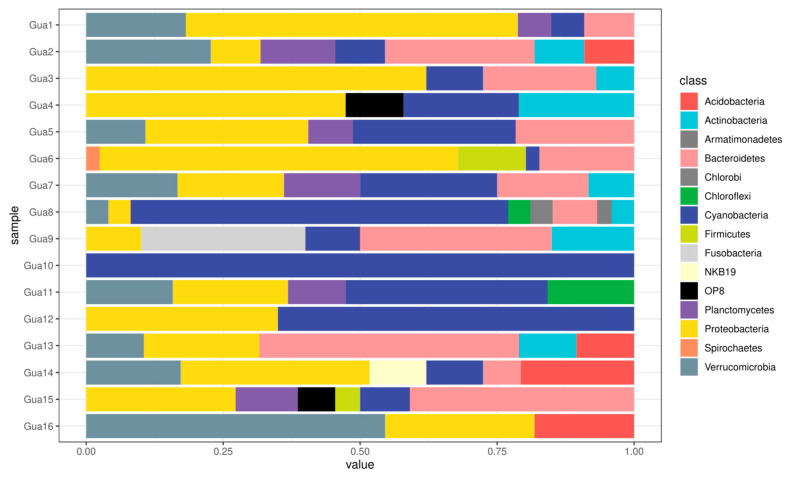
Phylogenetic analysis made through Qiime 2 software. The Proteobacteria phylum is predominant in 15 of the 16 sampling points.

On Qiime 2, the phylogenetic analysis revealed the presence of the phylum Proteobacteria at 15 sites, except at site Gua10, where there was an absolute 100% predominance of the phylum Cyanobacteria (Figure 6). The phylum Cyanobacteria was present at 14 of the 16 sites studied, apart from Gua10, with relative abundance at sites Gua8 (68.8%) and Gua12 (65%). The phylum Bacteroidetes was identified at 11 sites, with the highest proportion observed at Gua13 (47.3%). The phyla Verrucomicrobia and Planctomycetes were present at only nine and six sites, respectively, with the prominent presence of Verrucomicrobia at site Gua16, with a proportion of 54.54%.

### 3.5. Interaction Between Physical–Chemical Characteristics and Bacteria—An Obesrvation

The phylogenetic analysis of the bacterial data at phylum level, obtained using two different software programs, revealed similarity in composition, although the proportions showed distinct variations. The investigation of the bacterial composition through bacterial culture also highlighted that the *Proteobacteria* phylum was the only one identified in the bacterial culture studied. Table 3 shows the mean and standard error of the physicochemical parameters and the interaction with the bacterial culture test counts.

It was observed that by exclusively examining the sequencing results obtained from the two software packages and interacting them with the physicochemical characteristics of the first collection campaign from the surface of the reservoir, the *Firmicutes* phylum was detected only at Gua6 and Gua15 points, where ammonia values (4.1 mg/L) and phosphorus levels (1.1 mg/L and 1.2 mg/L, respectively) were similar. The significant presence of the *Chloroflexi* phylum was identified at points Gua8 and Gua11 only in the Qiime 2 software. In these cases, the water temperatures and pH values were remarkably close (22.5 °C and 21.4 °C for temperature, 7.2 and 7.3 for pH, respectively).

Additionally, in the Qiime 2 software, the predominance of the phyla *Chlorobi* and *Armatimonadetes* was only observed at point Gua8, where measurements of bathymetry, turbidity, dissolved oxygen (DO), pH, ammonia, nitrate, phosphorus and BOD were recorded as 1.6 m, 6.2 NTU, 8.9 mg/L, 7.2, 3.2 mg/L, 1.6 mg/L, 0.06 mg/L, and 5.3 mg/L, respectively.

On the other hand, points Gua8, Gua9, and Gua10 showed an absence of the phylum *Planctomycetes* on Qiime 2 and a very low frequency in EzBioCloud. However, it was not possible to discern a distinctly noticeable pattern of physico–chemical factors that could explain this absence.

**Figure 7:**
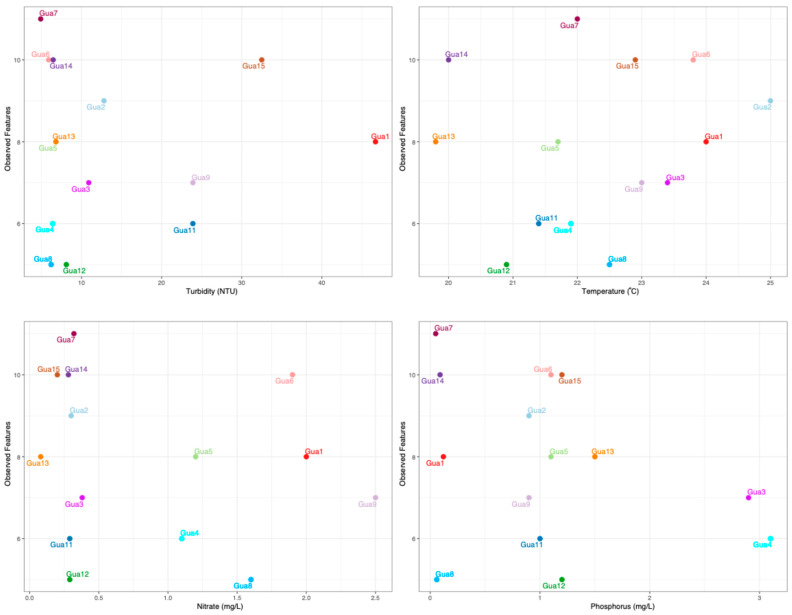

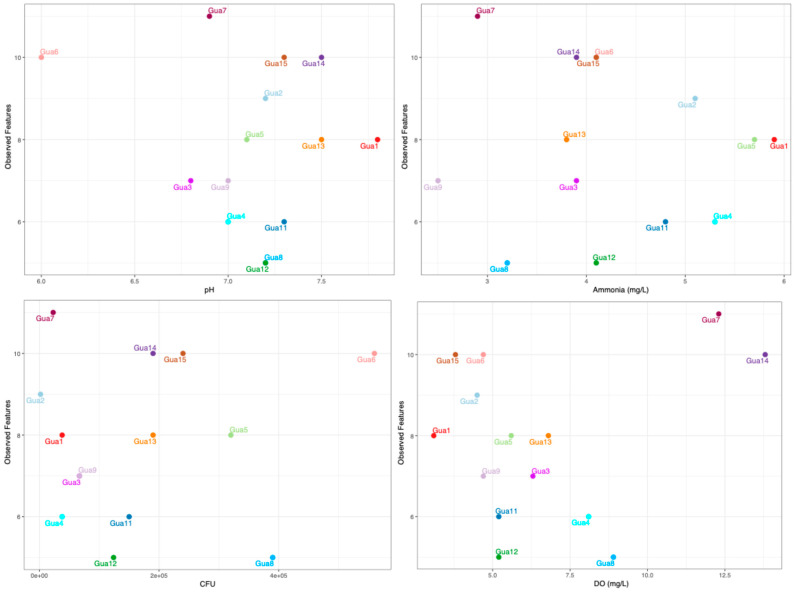
Interaction between the physicochemical data and Observed Features using the Qiime 2 software.

To further explore the data generated, we looked at the interaction between observed features and physicochemical parameters using Qiime 2 software, as shown in Figure 7. The Gua7 sample shows good quality in terms of dissolved oxygen, with ammonia and nitrate levels within acceptable limits. However, the high concentration of phosphorus suggests a risk of eutrophication, and the high CFU value indicates significant microbiological contamination. Despite this, the high number of observed features reflects a biodiverse environment, probably favored by the high level of oxygen.

The Gua8 sample has good levels of dissolved oxygen and pH within expectations, with ammonia and nitrate at safe levels. However, the phosphorus above the limit and the extremely high CFU value indicate serious contamination problems and ecological risk. The low number of observed features suggests a reduction in microbial diversity, possibly caused by contamination.

The Gua12 sample shows dissolved oxygen levels at the minimum limit, and ammonia is above the permitted values for the pH recorded, which indicates potential toxicity for many organisms. In addition, the high phosphorus and very high microbiological contamination (CFU) suggest a degraded environment with low capacity to support biodiversity. This is reflected in the low number of features observed.

Therefore, Gua7 is the most stable point, with good oxygen conditions, but is at risk of eutrophication and microbial contamination. Gua8 has adequate physical and chemical conditions, but bacterial contamination is affecting diversity, while Gua12 is in a critical state, with high levels of ammonia, phosphorus, and bacterial contamination, which compromises the local biodiversity.

### 3.6. Antimicrobial Resistance

A total of 30 genes related to antimicrobial resistance were investigated in the Guarapiranga reservoir. However, only 12 of them were detected in the samples analyzed: *ampC*, *bla_KPC_*, *bla_OXA-1_*, *bla_OXA-10_*, *bla_OXA-2_*, *bla_TEM_*, *intI*1, *sul1*, *sul2*, *tetA*, *tetB*, and *tetC*.

## 4. Discussion

### 4.1. Physicochemical

In this study, comprehensive analyses were carried out that included physicochemical assessments, as well as microbial composition profiles and the presence of ARGs in the Guarapiranga reservoir. This reservoir is located in the metropolitan region of São Paulo and plays a fundamental role as a significant source of supply for a population of more than 3 million individuals [8]. During the collections, it was possible to observe the occurrence of various indicators of contamination, including the presence of fecal material, as well as objects discarded in the reservoir, such as plastic bottles, clothes, and pieces of furniture. In addition, the expansion of irregular occupations and the malfunctioning of pumping stations were noted, contributing to the discharge of untreated sewage directly into the reservoir.

Since the late 1970s, the Environmental Company of the State of São Paulo (CETESB) has been continuously monitoring the quality of the water in the Guarapiranga reservoir [34] using a comprehensive set of parameters, including pH, turbidity, dissolved oxygen, total solids, biochemical oxygen demand (BOD), total phosphorus, total nitrogen, water temperature, and coliforms. Within this context, in the scope of this study, some parameters were selected for analysis, namely: temperature, turbidity, dissolved oxygen, BOD, pH, and phosphorus. However, total nitrogen was assessed indirectly through nitrate and ammonia concentrations in the water. In addition, it is important to note that total solids were not measured in this investigation, but the depth in meters of each collection point in the reservoir was recorded.

According to CONAMA Resolution 357/05 [35] the waters of the Guarapiranga reservoir are classified as Class 2, which means they are intended for uses such as human consumption, primary contact recreation (according to CONAMA Resolution 274 of the year 2000 [36]), aquaculture, fishing, irrigation of vegetables, and fruit plants. Consequently, the regulatory guidelines establish requirements such as the absence of color and odor; turbidity levels limited to 100 NTU; total phosphorus concentrations not exceeding 0.030 mg/L; the nitrate concentration must be a maximum of 10.0 mg/L; the concentration of total ammoniacal nitrogen must be a maximum of 3.7 mg/L N for pH less than or equal to 7.5, 2 mg/L N for pH greater than 5.5 and less than or equal to 8, 1 mg/L N for pH greater than 8 and less than or equal to 8.5, and 0.5 mg/L N for pH greater than 8.5; dissolved oxygen levels of no less than 5 mg/L O2; with regard to pH, variations in the 6.0 to 9.0 range are permitted; and biochemical oxygen demand (BOD) values over a five-day period at 20 °C must not exceed 5 mg/L O2. As for thermotolerant coliforms, the maximum permitted value is 1000 CFU.

In this study, all four samples from the surface layer showed turbidity levels significantly below 100 NTU; however, the average turbidity levels of the four samples from the bottom of the reservoir exceeded the established reference values. Regarding the dissolved oxygen (DO), only the first three sample collections from the reservoir bottom showed mean values below 5 mg/L O2. All four sample collections, both from the surface and the bottom of the reservoir, showed pH values within the reference ranges. However, the opposite is true for phosphorus levels in the reservoir. In addition, the color of the water varied from light/medium green to dark green/black.

According to the data presented by Semensatto et al. [34] over a period of 42 years of monitoring the waters of Guarapiranga, the average concentration of dissolved oxygen was 6.36 mg/L. In 2010, this average dropped to 5.57 mg/L, while during the present study it varied from 6.85 mg/L to 9.31 mg/L at the surface and from 4.18 mg/L to 7.36 mg/L in the deeper layers of the reservoir. Regarding total phosphorus, the average was 0.11 mg/L, with 2010 recording an average of 0.17 mg/L. Within the scope of this study, the averages of the surface samples ranged from 0.97 mg/L to 1.68 mg/L, while in the deeper layers of the reservoir these averages were between 2.63 mg/L and 4.45 mg/L. As for turbidity, the average over the 50-year study period was 17.3 mg/L, in contrast to the average of 11.62 mg/L in 2010. In this study, the averages of the surface samples ranged from 6.78 NTU to 27.79 NTU, while at depth they ranged from 139.20 NTU to 185.35 NTU. Regarding the pH, the average in previous years was 6.83, but in 2010 there was a greater variation, reaching a value of 7.1. Within the scope of this study, the averages of the surface samples ranged from 6.88 to 7.13, while in the deeper layers the variation was from 6.73 to 7.02.

This degradation of the reservoir was also observed by Bueno et al. (2020). In this study, they evaluated the water quality in fishing ponds located in the Guarapiranga sub-basin between November 2013 and August 2014, and it was possible to observe that the concentration of dissolved oxygen decreased and the levels of total phosphorus and total nitrogen increased in the effluents, exceeding the limits established by CONAMA resolution 357/2005 [8].

According to Fu et al. [37] ammonia has emerged as one of the main precursors of pollution in water intended for human consumption, deriving predominantly from anthropogenic activities, especially in urban contexts, thus consolidating itself as a highly significant indicator of water quality. Its potential is substantial, capable of delineating the presence of microorganisms and water contamination from sanitary effluents and animal waste, with the latter prevailing as the main source of contamination. Furthermore, in line with China’s GB5749-2006 standard [38], the parameters for ammonia levels are set at 0.5 mg/L, which is around seven times lower than the lowest average found at the bottom and eight times lower than the lowest average found on the surface of the Guarapiranga reservoir. In relation to the reference values stipulated by CONAMA, the concentration of ammonium nitrogen is higher than that stipulated in China, except for pH greater than 8.5, where the reference values are similar. That said, some points in Guarapiranga have ammonia concentrations within the reference values, for example, points Gua9 and Gua10 during the four collections. Regarding the average concentration, points Gua7 to Gua10, as well as the fourth collection, showed levels in line with the reference.

In this study of Guarapiranga, only one point, Gua7, during collections 3 and 4, showed BOD measurements within the reference values stipulated by CONAMA. BOD is a very important measure of water quality, as it indicates the amount of oxygen required for the decomposition of organic matter by microorganisms [39]. High BOD values can be interpreted as high organic pollution, which can negatively affect aquatic ecosystems and human health [40]. The importance of BOD is widely discussed in the literature, especially in the context of wastewater and natural waters. Recent studies have used machine learning techniques to predict BOD values in order to improve water quality management [39,41].

Fontana et al. (2014) outlined the history of the reservoir’s eutrophication, identifying significant ecological changes between 1919 and 2010. In this study, it was noted that the reservoir was classified as oligotrophic until 1947, became eutrophic around 1975 due to population growth, and has persisted in a state of severe eutrophication since 1990 [42]. The study conducted by Andrade et al. also showed that increasing urban occupation from the 1980s to 2012 negatively impacted the water quality of the reservoir when analyzing land use in the contributing watershed [43].

Therefore, through the physical–chemical analysis of the Guarapiranga water, it was possible to observe that many points during the four collection times showed indices that did not meet the reference values established by CONAMA, namely: 100% of the samples during the four collection campaigns from both the surface and the bottom of the reservoir showed phosphorus levels that exceeded the reference value; while for bottom turbidity, the percentage of samples that corresponded to the reference values varied from around 41.18% of the samples during the fourth collection to 94.12 during the third collection. Regarding the surface DO, samples outside the reference value ranged from 11.76% (third collection) to 31.25% (first collection), while at the bottom of the reservoir the range was 29.41% (fourth collection) to 70.59% (second collection). Regarding ammonia concentration, all the collection campaigns showed more than 50% of the samples outside the reference values: the lowest percentage of samples with ammonia concentration exceeding the reference values was observed during the fourth collection campaign of the surface samples (52.94%), while the highest percentage of samples was recorded during the third collection campaign of the samples from the bottom of the reservoir. About the pH, few samples exceeded the reference values: only sample Gua10 during the second collection campaign from the surface, corresponding to 5.88%, while at the bottom of the reservoir it was observed that most of the samples that showed values outside the reference were found during the third collection, corresponding to 11.8%. As for BOD, the samples outside the norm ranged from 94.12% during the third and fourth collection campaigns to 100% during the first and second collection campaigns.

However, it was not possible to find a pattern in the changes observed, nor an association with the points collected. However, it is worth highlighting the Gua7 surface point, as it did not exceed the reference values for DO, pH and nitrate, and only exceeded the reference value for ammonia during the third collection (which represents only 25%), as well as being the only point to have BOD values in accordance with the reference, i.e., 50% of the samples collected at this point showed results in accordance with CONAMA legislation. Gua7 is close to the Sacred Ground of Guarapiranga—World Messianic Church of Brazil, where public access to the reservoir is more restricted. In addition, during the collections it was also observed that the color of the water varied from light/medium green to dark green/black.

Regarding the coliforms, this study showed that not only *E. coli* was found in Guarapiranga, but also *Salmonella* spp., *Shigella* spp., and *Klebsiella* spp. in most of the sampling points collected during the four samples, which may be associated with the contamination of the reservoir by sewage [36]. In addition, according to Brazilian legislation, 1000 CFUs is recommended. In this study, point Gua16 had the lowest count during the fourth surface collection of 1000 CFUs, while point Gua8 had the highest count, 5,300,000 CFUs, during the fourth collection from the bottom of the reservoir.

Recent studies have made the association between altered physical and chemical properties of water and public health. These articles reveal the health risks arising from changes in water quality influenced by various environmental and anthropogenic factors. For example, increased levels of nitrates and phosphates in water can lead to eutrophication, impacting human health through algal toxins [44]. A study carried out in Brazil showed that high levels of phosphorus and thermotolerant coliforms in river basins were associated with outbreaks of gastrointestinal diseases, highlighting the need for integrated analysis of qualitative and quantitative water data [45].

Therefore, the pollution and degradation of the Guarapiranga reservoir, which has been occurring over the years and is becoming more and more pronounced, violates the Brazilian Article 35 of Law 9866 of 28 November 1997, since such pollution, especially by coliforms, has serious consequences for public health.

### 4.2. Bacteriome Diversity and Composition

Among the bacteria found in the aquatic environment, the most common include the *Proteobacteria*, *Actinobacteria*, *Cytophaga-Flavobacteria-Bacteroidetes* (CFB), *Cyanobacteria*, and *Verrucomicrobia* and, among these groups, the *Betaproteobacteria* are frequently studied and can be abundant in epilimnetic waters, i.e., the upper layer of the reservoir, representing up to 60–70% of the total bacterial cells. These bacteria are influenced by high concentrations of dissolved organic carbon and nitrate, and are often associated with cyanobacteria or particles of different sizes [10].

The analysis of the sequencing data by both the EzBioCloud and Qiime 2 tools revealed the presence of *Proteobacteria*, *Cyanobacteria*, *Bacteroidetes*, *Verrucomicrobia*, and *Planctomycetes*. However, the relative frequency of the phyla varied between the tools, quite possibly due to the regions of the bacterial genome analyzed. Similarly to this study, Soares et al. [46] in their work published in 2021, also identified the phyla *Bacteroidetes*, *Proteobacteria*, *Verrucomicrobia*, and *Planctomycetes* as the most abundant in their work carried out in Guarapiranga. This similarity was maintained even though they used different reagents and methods, such as a different extraction kit, library preparation and sequencing platform compared to this work.

However, when comparing the results of the same sequences generated but analyzed in the different software used in this study, there were variations in the presence and proportion of these phyla. The *Proteobacteria* phylum emerged as prevalent at all collection points in EzBioCloud, but Qiime2 analysis indicated its absence at point Gua10. The Cyanobacteria phylum also showed contrasting patterns, with 100% prevalence in Gua10 by Qiime2 and 20.88% in Gua9 by EzBioCloud. These discrepancies in results could be attributed, in part, to the different taxonomic databases and 16S rRNA gene region segments used by each software. In addition, perhaps the choice of the V3/V4 versus V4 region could lead to variations in the ability to identify and quantify certain bacterial phyla. In addition, there is the hypothesis that the variability in the composition of the bacteriome may be influenced by environmental factors specific to each collection point, adding complexity to the interpretation of the results. However, it is worth noting that, regardless of the differences between the software platforms, the *Proteobacteria* phylum remained a prominent bacterial component at all sampling sites, reflecting its adaptation and prevalence in this aquatic environment.

### 4.3. Intereation Between Physicochemical Characteristics and Bacterial Composition

Several studies have shown that physicochemical variables such as temperature, photoperiod, and nutrient concentration influence the variability of bacterial communities in aquatic environments [47,48,49], with temperature possibly being the most important abiotic factor for the growth and activity of the microbial population [48]. Another widely recognized factor is the presence of phosphorus, one of the essential elements for cell development and functionality. A shortage of this element can impact the behavior of microorganisms at both the cellular and community level, since a lack of phosphorus is associated with a decrease in the microbial community and can also activate a lethal phenotype in *Pseudomonas aeruginosa* [49]. Therefore, establishing an understanding of the relationship between water quality parameters and bacterial composition is fundamental to tracking changes in the microbiome through these indicators [50].

Li et al. [50] made relevant observations regarding the differential distribution of bacterial phyla in relation to environmental factors. They reported that the predominant presence of *Alphaproteobacteria* is associated with higher temperatures and lower levels of turbidity. On the other hand, a possible positive relationship between high levels of turbidity and the presence of ammonia was identified with the *Actinobacteria* phylum. In addition, the *Planctomycetes* and *Verrucomicrobia* phyla showed greater prevalence in environments characterized by high turbidity and high ammonia concentrations. On the other hand, *Cyanobacteria* seem to be favored by lower temperatures and higher levels of dissolved oxygen. However, it is interesting to note that the observations by Thomas and Litchman [51] show opposite results regarding the relationship between temperature and the predominance of *Cyanobacteria*. This discrepancy could be attributed to the diversity of *Cyanobacteria* species, each exhibiting a specific adaptation to different temperature ranges.

In this study, using the EzBioCloud tool, it is worth highlighting the identification of the presence of *Cyanobacteria* at the Gua9 collection point, with a significant prevalence of 20.88%. The environmental conditions recorded at this point included a temperature of 23 °C, a pH of 7, a dissolved oxygen (DO) level of 4.7 mg/L, a phosphorus concentration of 0.9 mg/L, as well as ammonia and nitrate levels of 2.5 mg/L. In addition, water turbidity was measured at 23.9 NTU. On the other hand, the analysis conducted using the Qiime 2 software revealed the remarkable prevalence of the *Cyanobacteria* phylum in Gua10, with a predominance of 100%. The environmental conditions at this point were characterized by a turbidity of 28.1 NTU, temperature of 23.4 °C, DO of 10.8 mg/L, pH of 7, ammonia concentration of 2.1 mg/L, nitrate of 0.4 mg/L, and phosphorus of 1 mg/L. Additionally, at point Gua16, analysis also by Qiime 2 identified the phylum *Verrucomicrobia* with a significant prevalence of 54.54%. At this site, the parameters recorded included a turbidity of 14.8 NTU, temperature of 20.8 °C, DO of 5.8 mg/L, pH of 7.3, ammonia concentration of 3.8 mg/L, nitrate of 0.21 mg/L, and phosphorus of 0.9 mg/L.

In relation to total phosphorus, it is known that its accurate monitoring is crucial for the management of aquatic environments and the control of eutrophication [52], since the process of eutrophication can restrict the use of water for fishing, recreation, and industry due to the increased growth of undesirable algae and aquatic plants, as well as the shortage of oxygen caused by the death and decomposition of these plants, which can result in the harmful proliferation of algae and compromised water quality [53]. It is also known that increasing the concentration of trace phosphorus in drinking water can lead to an increase in microbial biomass and the concentration of opportunistic pathogens, affecting water quality [54].

In the study conducted by Xie et al., it was noted that the amount of phosphorus-solubilizing bacteria, which belong to groups such as *Proteobacteria* (including, for example, the genus *Pseudomonas*), increases as the concentration of water-soluble phosphorus in the soil increases [55]. However, when examining the relationship between *Proteobacteria* and phosphorus in water, Liang et al. observed a negative correlation between *Proteobacteria* and both organic phosphorus and sulfide ions, while finding a positive association with residual phosphorus [56].

In the Billings reservoir, also in the city of Sao Paulo, Brazil, the study conducted by Marcondes et al. revealed an abundance of the *Cyanobacteria* phylum, followed by *Proteobacteria*, and showed that phosphorus concentration was one of the factors that most influenced the composition of the bacterial community [24]. When studying the Pinheiros River, a heavily polluted river in the city of São Paulo, Brazil, Godoy et al. detected the phyla *Proteobacteria*, *Firmicutes*, and *Bacteroidetes* as the most abundant, which were also detected in domestic sewage sludge from São Paulo. In addition, this work showed that certain environmental variables, such as phosphate, ammonium nitrate and DO, were considered important factors that structured bacterial communities within this ecosystem [57]. The results obtained in the Guarapiranga reservoir show the phosphorus concentration exceeded 100% of the sample points collected and the *Proteobacteria* phylum was present in high relative frequency at all points by analyzing the data generated by sequencing the 16S gene. In addition, *Pseudomonas* spp. was also found at point Gua5 by using bacterial culture. In addition to *Proteobacteria*, *Cyanobacteria*, and *Bacteroidetes* were also detected in the Guarapiranga reservoir, as well as in Billings reservoir and Pinheiros River [24,57].

### 4.4. Antibiotic Resistance Analysis

Antibiotic-resistance genes (ARGs) have emerged as a component of environmental pollution of significant relevance, with resistance genes to tetracyclines and sulfonamides standing out as the most prevalent in the environment [58]. An investigation conducted in China revealed the presence of ARGs in wastewater, [59] given that conventional treatment processes are not specifically designed for the elimination of genetic material [60]. As elucidated by Le-Minh, Khan, and Drewes [61], the presence of antibiotics in sewage can be attributed to human excretion, since several active antibiotics are not completely metabolized during therapeutic use and are excreted in the sewage in their original form. In addition, the improper disposal of unused medicines into the wastewater system and their use in veterinary medicine also contribute to the presence of antibiotics in these waters [61].

In this context, among the 30 resistance genes analyzed in Guarapiranga, it was possible to detect the presence of 12 of them: AmpC, bla_KPC_, blaTEM, blaOxa-1, blaOXA-2, blaOXA-10, int1, Sul1, Sul2, tetA, tetB, and tetC.

As indicated by Gillings et al. [60] bacterial abundance and, consequently, the presence of the intl1, or int-1, gene, can show an increase during the wastewater treatment process. This phenomenon occurs because bacteria carrying class 1 integrons associated with resistance factors, or those capable of acquiring them through lateral gene transfer, can experience an increase in their abundance throughout various stages of the wastewater treatment process.

In addition, according to the work published by Leng et al. in 2020, class 1 integrons can reflect the abundance of ARGs and can be considered an indicator of anthropogenic pollution. Such ARGs can be transferred to human pathogens through the facilitating functions of integrons, which represents a potential crisis for human health [58]. Understanding and controlling the spread of integrons and ARGs is crucial for the sustainable management of water resources and the protection of human health, requiring strategies that encompass improvements in wastewater treatment practices and continuous monitoring of water quality.

The AmpC gene refers to the AmpC beta-lactamase gene, which can be intrinsic in some Gram-negative bacteria, such as *Citrobacter* spp., *Enterobacter* spp., *Serratia* spp. and *Morganella* spp. and can be expressed constitutively or induced. This gene has also been identified in plasmids present in isolates of *K. pneumoniae* and *E. coli*. The detection of the AmpC gene in the waters of Guarapiranga shows a direct correlation with the microbiological findings, showing the presence of *E. coli* at all the points sampled, as well as *Klebsiella* spp. at most of the points. Furthermore, AmpC beta-lactamase is recognized as one of the main mechanisms underlying resistance to broad-spectrum cephalosporins [62].

The bla_KPC_ gene, encoding a broad-spectrum carbapenemase capable of hydrolyzing various beta-lactams, including carbapenems, was first identified in an isolate of *Klebsiella* pneumoniae in the United States, a study of which was published in 2001 [63]; the beta-lactamases blaTEM, widely distributed in Gram-negative bacteria such as *Enterobacteriaceae* [64], and the extended-spectrum beta-lactamases blaOxa-1 and blaOXA-10, [65], have been detected in isolates of *Klebsiella* sp. and the former also in *E. coli* [66]. In addition, the blaOXA-2 gene was also present in Guarapiranga. This set of resistance genes is correlated with the bacterial species identified in the microbiological analyses.

The tetA, tetB, and tetC genes, also identified as positive in this study, are tetracycline-resistance genes often found in high frequency in Gram-negative bacteria, including *E. coli* [67]. On the other hand, the two sulfonamide-resistance genes, Sul1 and Sul2, detected in Guarapiranga, have significant implications in the context of the development of antibiotic-resistant bacteria. According to Suzuki et al., sulfonamides are known for their low affinity for forming complexes with metal ions, high water solubility and stability, which can preserve their activity against bacteria when introduced into the aquatic environment, due to their chemical characteristics. In addition, these substances have the potential to accelerate the emergence of antibiotic-resistant bacteria in natural microbial communities [68].

Tetracycline and sulfonamide residues that enter the aquatic environment can pose a considerable ecological risk. These antimicrobials are often detected in wastewater treatment plant effluents, agricultural runoff, and landfill leachate. Once in the aquatic environment, they can persist and influence microbial ecology [69]. Studies show that these antimicrobials can have acute and chronic toxicity in aquatic organisms such as fish, micro-crustaceans and algae. For example, neomycin, an aminoglycoside, has been shown to be highly toxic to Daphnia magna, while sulfamethoxazole, a sulfonamide, and oxytetracycline, a tetracycline, showed intermediate toxicity [70]. Although some antimicrobials are degraded through biological and photochemical processes, many of them can bind to sediment particles, prolonging their stay in the environment. In the case of tetracyclines, for example, they have a high affinity for solid particles, which can hinder their complete degradation [71].

Leng et al. identified a positive correlation between the Sul2 and NH3-N. This is because NH3-N tends to react with oxidation factors in the environment, which in turn reduces the effectiveness of ARG removal by these oxidation processes. In addition, the increase in microbial mass resulting from the utilization of NH3-N by microorganisms in the sediment leads to an increase in ARG levels [58].

In addition to that, Leng et al. also found a positive correlation between Sul2 genes and total phosphorus. Similarly, phosphorus, as a nutrient, has the ability to increase the biomass of microorganisms that carry antibiotic-resistance genes [58].

In relation to the Guarapiranga reservoir, in addition to the detection of ARGs by this study, the study by Shihomatsu et al. (2017) highlighted the presence of pharmaceutical compounds and illicit drugs in the reservoir, which indicates the pollution of the reservoir by raw sewage and urban drainage [71].

## 5. Conclusions

This study highlights the progressive decline in water quality in the Guarapiranga reservoir due to the illegal dumping of sewage, possibly caused by an increase in nearby irregular dwellings. The study used next-generation sequencing to analyze the microbial diversity present in the reservoir and found variations in the relative frequency of different bacterial phyla. However, the overall results were consistent, indicating that a combined bioinformatics approach can provide a comprehensive understanding of the microbial diversity. Additionally, the study detected the presence of antibiotic-resistance genes in the reservoir, raising concerns about the spread of antimicrobial resistance in the aquatic environment.

The combination of physicochemical results, coliforms, and ARGs obtained in this work show that the reservoir has suffered a progressive reduction in water quality, largely due to the illegal dumping of sewage into the waters of Guarapiranga as a result of the increase in the number of irregular dwellings in the vicinity.

Analysis of the sequencing data using both the EzBioCloud tool and Qiime 2 revealed the presence of *Proteobacteria*, *Cyanobacteria*, *Bacteroidetes*, *Verrucomicrobia*, and *Planctomycetes*. However, the relative frequency of the phyla varied between the tools, quite possibly due to the regions of the bacterial genome that were analyzed. Still in relation to sequencing, a limitation of the study lies in the fact that only the surface samples collected during the first collection were sequenced, making it impossible to carry out comparative analyses of the microbiome at different times in the reservoir.

These findings emphasize the need for improved public and environmental policies in the Guarapiranga reservoir region. Based on the findings of this study and in accordance with CONAMA regulations, it is recommended that the waters of the Guarapiranga reservoir be reclassified, as the reservoir’s waters are currently not in the best condition for human consumption, aquaculture, fishing, and irrigation. Further research is necessary to continue monitoring the reservoir’s water quality.

## Figures and Tables

**Table 1:** Studied ARGs, with their related resistances.

Genes	Resistance	Genes	Resistance
aacC2	Aminoglycosides	int1	Various antibiotics (presence of integrons)
aacC3	Aminoglycosides	qepA	Fluoroquinolones
aacC4	Aminoglycosides	qnrA	Fluoroquinolones
AmpC	Beta-lactams	qnrB	Fluoroquinolones
blaCMY	Beta-lactams	qnrD	Fluoroquinolones
blaDHA	Beta-lactams	qnrS	Fluoroquinolones
blaGes	Beta-lactams	Sul1	Sulfonamides
blaKPC	Beta-lactams *	Sul2	Sulfonamides
blaNDM	Beta-lactams *	Sul3	Sulfonamides
blaOxa-1	Beta-lactams	ermC	Macrolides (erythromycin)
blaOxa-10	Beta-lactams	tetA	Tetracyclines
blaOxa-2	Beta-lactams	tetB	Tetracyclines
blaSHV	Beta-lactams	tetC	Tetracyclines
blaTEM	Beta-lactams	tetD	Tetracyclines
blaVIM	Beta-lactams *	tetM	Tetracyclines

* beta-lactams, including carbapenems.

**Table 2:** Results of microbiological cultures conducted at 17 points during the four collection campaigns (sample was not collected at point Gua17 during the first collection).

Samples	Genus/Species
Gua1	*Salmonella* spp., *Shiguella* spp. and *Escherichia coli*
Gua2	*Salmonella* spp., *Shiguella* spp., *Klebsiella* spp. and *Eschericha coli*
Gua3	*Salmonella* spp., *Shiguella* spp., *Klebsiella* spp. and *Eschericha coli*
Gua4	*Salmonella* spp., *Shiguella* spp., *Klebsiella* spp. and *Eschericha coli*
Gua5	*Salmonella* spp., *Pseudomonas* spp., *Shiguella* spp. and *Escherichi coli*
Gua6	*Escherichia coli* e *Salmonella* spp.
Gua7	*Salmonella* spp., *Shiguella* spp., *Klebsiella* spp. and *Eschericha coli*
Gua8	*Salmonella* spp., *Shiguella* spp., *Klebsiella* spp. and *Eschericha coli*
Gua9	*Salmonella* spp., *Shiguella* spp., *Klebsiella* spp. and *Eschericha coli*
Gua10	*Salmonella* spp., *Shiguella* spp., *Klebsiella* spp. and *Eschericha coli*
Gua11	*Salmonella* spp., *Shiguella* spp., *Klebsiella* spp. and *Eschericha coli*
Gua12	*Salmonella* spp., *Shiguella* spp. and *Escherichia coli*
Gua13	*Salmonella* spp., *Shiguella* spp. and *Escherichia coli*
Gua14	*Salmonella* spp., *Shiguella* spp. and *Escherichia coli*
Gua15	*Salmonella* spp., *Shiguella* spp., *Klebsiella* spp. and *Eschericha coli*
Gua16	*Salmonella* spp., *Shiguella* spp. and *Escherichia coli*
Gua17	*Salmonella* spp., *Shiguella* spp., *Klebsiella* spp. and *Eschericha coli*

**Table 3:** Interaction between Physicochemical Characteristics and Bacterial Counts. For each column representing the physicochemical parameters and CFU, numbers 1 and 2 correspond to the mean of the four collection campaigns and the standard error, respectively. The reference values according to Brazilian Environment National Council (CONAMA) Resolution 357/05 are as follows: Turbidity: up to 100 NTU; Dissolved Oxygen (DO): minimum of 5 mg/L; pH: 6.0 to 9.0; Total Ammoniacal Nitrogen: for pH ≤ 7.5–3.7 mg/L N, for 7.5 < pH ≤ 8.0–2.0 mg/L N, for 8.0 < pH ≤ 8.5–1.0 mg/L N, for pH > 8.5–0.5 mg/L N; Nitrate: up to 10 mg/L; Phosphorus: up to 0.030 mg/L; Biochemical Oxygen Demand (BOD) for 5 days at 20 °C: up to 5 mg/L O_2_.

Sample (Bottom)	Depth (Meter)		Turbidity (NTU)		Water Temperature (°C)		DO (mg/L)		pH		Ammonia (mg/L)		Nitrate (mg/L)		Phosphorus (mg/L)		CFU	
	1	2	1	2	1	2	1	2	1	2	1	2	1	2	1	2	1	2
Gua1	1.40	0.39	108.08	30.00	21.83	0.96	5.10	1.01	7.05	0.37	6.28	0.28	1.93	0.69	11.66	0.50	30,750.00	10,734.48
Gua2	2.83	0.43	334.75	208.48	21.75	0.51	6.60	2.26	6.60	0.11	4.20	0.35	0.20	0.04	2.21	0.26	9725.00	4712.28
Gua3	1.50	0.25	44.08	14.64	21.60	0.66	6.80	1.18	7.13	0.08	5.75	1.21	0.62	0.04	2.02	0.32	61,175.00	18,529.63
Gua4	1.23	0.20	97.98	7.28	21.60	0.56	6.63	4.53	6.90	0.07	5.50	0.19	1.05	0.06	3.05	0.05	72,000.00	13,335.42
Gua5	1.15	0.06	144.61	46.18	21.33	0.64	5.25	1.64	6.95	0.03	5.43	0.22	3.65	0.81	4.37	2.40	576,000.00	107,063.84
Gua6	1.90	0.09	215.85	96.87	21.35	0.61	2.00	0.60	6.00	0.07	4.85	0.39	2.07	0.27	6.33	0.25	696,500.00	107,698.27
Gua7	4.13	0.56	147.60	26.27	21.05	0.53	7.18	1.31	6.68	0.27	3.70	0.11	0.89	0.04	0.85	0.56	23,500.00	3662.88
Gua8	2.25	0.55	198.00	29.45	20.55	0.68	5.80	1.43	6.78	0.13	1.70	0.39	0.90	0.25	0.67	0.53	1,792,500.00	1,171,589.91
Gua9	2.58	0.63	117.28	42.60	21.08	0.63	4.45	0.36	6.78	0.23	4.70	0.32	2.58	0.23	3.72	2.29	62,500.00	8490.19
Gua10	2.68	0.48	189.15	55.76	21.25	0.75	4.73	0.17	6.38	0.22	3.88	0.13	0.42	0.14	1.97	0.30	689,250.00	182,758.21
Gua11	4.70	0.56	141.68	41.01	20.53	0.50	4.43	0.37	6.90	0.18	5.18	0.29	0.34	0.02	1.74	0.33	178,000.00	4546.06
Gua12	2.00	0.38	187.65	68.13	20.05	0.53	4.58	0.67	7.48	0.51	4.58	0.23	0.34	0.02	6.96	1.18	202,500.00	23,935.68
Gua13	6.63	0.84	92.45	24.20	20.33	0.34	5.38	0.22	7.20	0.14	4.08	0.09	0.10	0.00	1.97	0.05	223,750.00	14,343.26
Gua14	7.93	1.52	268.93	178.84	21.08	0.55	6.45	1.39	7.10	0.11	4.13	0.06	0.29	0.04	1.84	0.48	228,250.00	12,769.59
Gua15	1.38	0.14	240.15	78.80	20.68	0.49	3.03	1.14	7.10	0.07	4.33	0.12	0.25	0.01	1.59	0.16	276,250.00	11,433.69
Gua16	2.20	0.47	94.43	33.51	20.18	0.42	4.29	0.51	7.15	0.06	3.88	0.27	0.21	0.03	1.34	0.19	62,800.00	46,121.29
Gua17	1.70	0.60	123.00	8.96	19.20	0.49	3.90	0.91	6.77	0.19	3.97	0.09	1.17	0.18	1.58	0.23	254,666.67	8666.67
**Sample (Surface)**	**BOD** **(mg/L)**		**Turbidity (NTU)**		**Water** **temperature (°C)**		**DO** **(mg/L)**		**pH**		**Ammonia (mg/L)**		**Nitrate (mg/L)**		**Phosphorus (mg/L)**		**CFU**	
	**1**	**2**	**1**	**2**	**1**	**2**	**1**	**2**	**1**	**2**	**1**	**2**	**1**	**2**	**1**	**2**	**1**	**2**
Gua1	7.18	0.45	23.23	9.33	22.88	0.53	6.43	1.16	7.43	0.31	5.25	0.23	1.42	0.52	0.27	0.06	27,850.00	9727.41
Gua2	6.25	0.19	14.62	4.45	22.65	0.92	6.88	1.15	6.85	0.23	4.23	0.30	0.20	0.07	0.79	0.21	7725.00	3942.16
Gua3	8.20	0.37	12.88	6.14	21.80	0.94	9.10	1.91	6.93	0.21	4.68	1.05	0.46	0.06	1.72	0.49	52,075.00	15,370.61
Gua4	8.10	0.18	12.12	5.29	21.78	0.78	9.98	3.28	6.53	0.22	5.03	0.11	0.90	0.07	2.85	0.09	44,000.00	3341.66
Gua5	6.75	0.19	14.38	5.54	21.45	0.70	7.10	1.12	6.95	0.10	4.93	0.35	0.94	0.15	2.02	1.08	556,500.00	103,628.74
Gua6	8.88	0.21	4.51	0.53	22.20	1.02	4.90	1.08	6.15	0.12	4.40	0.40	1.43	0.25	1.14	0.22	565,000.00	82,209.08
Gua7	4.98	0.41	16.40	6.94	21.55	0.80	8.90	1.53	6.40	0.19	3.25	0.23	0.30	0.02	0.78	0.71	18,500.00	1707.83
Gua8	5.60	0.21	10.84	5.46	22.10	0.96	7.65	1.38	7.13	0.05	3.04	0.27	1.46	0.30	0.65	0.56	127,975.00	88,544.34
Gua9	5.90	0.25	15.87	5.88	22.18	0.73	6.60	1.14	6.98	0.05	2.20	0.13	1.30	0.42	0.91	0.34	44,750.00	9953.01
Gua10	6.35	0.21	15.00	7.42	21.98	0.78	10.38	2.06	6.55	0.35	2.20	0.21	0.64	0.12	1.07	0.14	440,000.00	125,233.12
Gua11	6.08	0.42	16.14	5.72	21.10	0.50	6.93	1.81	7.15	0.22	4.75	0.17	0.25	0.02	1.07	0.14	162,000.00	4546.06
Gua12	7.08	0.28	11.96	6.78	21.33	0.25	6.20	0.53	7.48	0.23	4.10	0.29	0.25	0.02	1.76	0.29	127,000.00	38,594.04
Gua13	8.05	0.30	13.00	5.16	21.40	0.54	9.65	1.09	7.60	0.14	3.95	0.06	0.08	0.00	1.60	0.10	206,750.00	12,202.29
Gua14	6.15	0.31	12.04	5.27	21.33	0.47	12.60	0.65	7.23	0.11	3.80	0.14	0.29	0.01	0.42	0.34	191,500.00	4272.00
Gua15	11.13	0.77	19.39	7.02	21.68	0.48	4.33	1.74	7.18	0.11	4.20	0.24	0.19	0.00	1.23	0.08	236,250.00	5543.39
Gua16	8.03	0.18	13.93	5.13	20.75	0.38	5.68	0.87	7.48	0.11	3.58	0.28	0.18	0.03	1.06	0.06	55,250.00	41,825.78
Gua17	8.30	0.21	15.48	11.39	21.17	1.30	6.50	2.40	7.37	0.09	3.93	0.09	1.07	0.12	1.25	0.46	233,333.33	14,529.66

## Data Availability

DOI for the Fastq and Qiime 2: 10.5281/zenodo.13776935.

## Supplementary Materials

The following supporting information can be downloaded at: https://www.mdpi.com/article/10.3390/life15050729/s1, Table S1: Coordinates; Table S2: Bottom; Table S3: Surface; Table S4: Seq EzBioCLoud; Table S5: Seq Qiime2.
